# Increased visceral fat area to skeletal muscle mass ratio is positively associated with the risk of cardiometabolic diseases in a Chinese natural population: A cross‐sectional study

**DOI:** 10.1002/dmrr.3597

**Published:** 2022-12-02

**Authors:** Shi Zhang, Yaping Huang, Jing Li, Xincheng Wang, Xiaohe Wang, Minying Zhang, Yanju Zhang, Meiyang Du, Jingna Lin, Chunjun Li

**Affiliations:** ^1^ Department of Endocrinology Health Management Center Tianjin Union Medical Center Nankai University Affiliated Hospital Tianjin China; ^2^ Tianjin Centers for Disease Control and Prevention Tianjin China; ^3^ School of Medicine Nankai University Tianjin China

**Keywords:** cardiometabolic diseases, gender difference, obesity, skeletal muscle mass, visceral fat area

## Abstract

**Aims:**

Visceral adiposity and skeletal muscle loss may be positively correlated with cardiometabolic outcomes. This study aimed to explore the associations between the visceral fat area to skeletal muscle mass ratio (VSR) and the risk of cardiometabolic diseases in a Chinese natural population.

**Materials and Methods:**

A total of 5158 participants were included in this study. Body composition, anthropometrical, and biochemical measurements were performed. Body composition was assessed via the direct segmental multi‐frequency bioelectrical impedance analysis method. The associations between VSR and metabolic associated fatty liver disease (MAFLD), hyperglycemia, hypertension, dyslipidemia, and hyperuricemia were analysed.

**Results:**

With the increase of VSR by one quartile, the odds ratio (OR) increased significantly for all five cardiometabolic diseases in both genders (*p*_trend_ < 0.001). With regard to the highest versus the lowest quartile of VSR, the ORs for cardiometabolic diseases were significantly higher in women than in men. Restricted cubic splines showed that there were significant non‐linear relationships between VSR and the risk of MAFLD, dyslipidemia, hyperglycemia, and hypertension in both genders (*p* for non‐linearity <0.05). The risk was relatively flat until VSR reached 3.078 cm^2^/kg in men and 4.750 cm^2^/kg in women and started to increase rapidly afterwards. In men, however, the risk slowed down after the VSR value reached around 4 cm^2^/kg.

**Conclusions:**

VSR was positively associated with cardiometabolic diseases regardless of gender. As VSR increased, the risk of cardiometabolic diseases was significantly higher in women than in men.

**Trial Registration:**

www.chictr.org.cn (Registration number: ChiCTR2100044305).

## 1 INTRODUCTION

The prevalence of obesity has increased dramatically worldwide in the past few decades. Obesity is considered a risk factor for cardiometabolic diseases such as metabolic associated fatty liver disease (MAFLD), type 2 diabetes mellitus (T2DM), hypertension, dyslipidemia, and hyperuricemia.^1^ Obesity and its closely associated cardiometabolic disorders have placed a heavy burden on the public health system globally.^2^ Since body mass index (BMI) is the most easily measured parameter of obesity, it is widely used for the assessment of obesity in clinical practice. However, BMI fails to accurately distinguish between body fat mass and lean mass, and moreover, it cannot describe the distribution of body fat. Mounting evidence suggests that visceral fat deposition is the main cause for the development of cardiometabolic diseases, whereas subcutaneous fat accumulation may be associated with a decreased risk of the aforementioned cardiometabolic conditions.^3^ Consequently, studies that do not take abdominal obesity into consideration and use BMI to define obesity have most commonly demonstrated the ‘obesity paradox’.^4^ In addition to visceral adiposity, the impact of reduced skeletal muscle mass on the development of insulin resistance, T2DM, and other cardiometabolic outcomes has become the focus of attention in recent years.^5^

Increased visceral fat and decreased skeletal muscle mass have both been associated with an increased risk of cardiometabolic disease; therefore, they may have a synergistic effect on cardiometabolic disorders.^6^ What's more, it is generally recognised that there exist gender differences in fat distribution. Men are more prone to have central obesity due to the expansion of visceral fat deposition, whereas women tend to be ‘pear‐shaped’, with preferential deposition of fat in the lower body, particularly in the glutaeal‐femoral region.^7^ To date, however, there have been no large‐scale studies investigating the combined effect of increased visceral fat and decreased skeletal muscle mass on cardiometabolic outcomes in natural populations. And whether the joint effect of these two distinct compartments of body composition differs between different genders is unknown yet. Therefore, this study aimed to explore the associations between the visceral fat area to skeletal muscle mass ratio (VSR) and the risk of several cardiometabolic diseases, including MAFLD, hyperglycemia, hypertension, dyslipidemia, and hyperuricemia, in a Chinese population undergoing regular health check‐ups and further elucidate the difference in this association between men and women.

## 2 MATERIALS AND METHODS

### 2.1 Study design and sample collection

**FIGURE 1:**
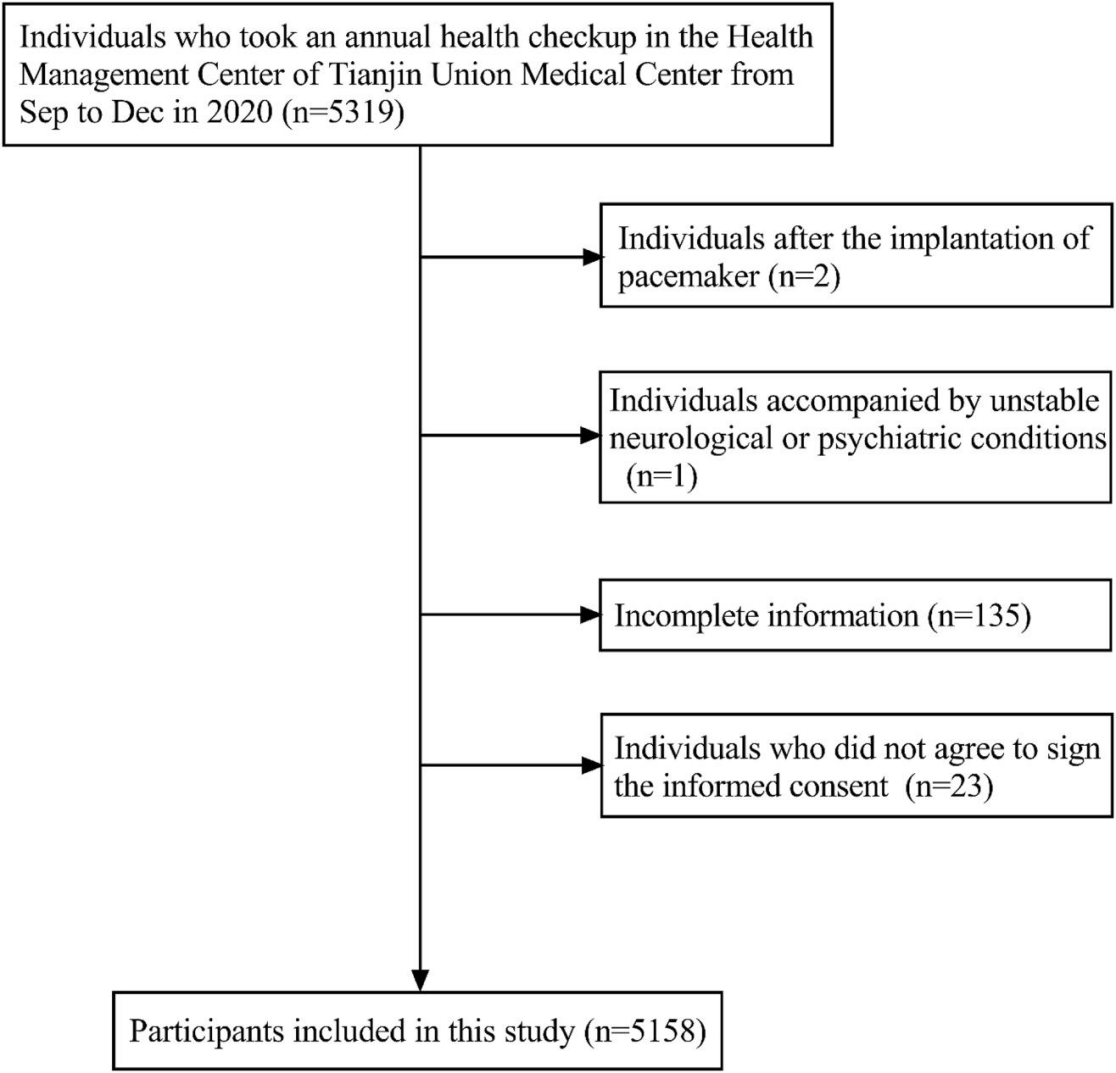
Study flow chart illustrated the flow of the participants included in this study.

The group who took an annual health check‐up in the Health Management Center of Tianjin Union Medical Center from September to December in 2020 was our target population. The total number of people during this period was 5319. Individuals were excluded if they were after the implantation of a pacemaker, accompanied by unstable neurological or psychiatric conditions, unwilling to sign the informed consent, or if their information was incomplete. Finally, a total of 5158 participants were included in this study after 161 were excluded. The sample collection process is shown in Figure 1. The Medical Ethics Committee of Tianjin Union Medical Center (No. 2021C06) approved the study, and written informed consents were provided by all the participants.

### 2.2 Anthropometrical measurement and body composition evaluation

After an overnight fast, all participants were instructed to wear light clothing without shoes and perform anthropometrical evaluations, including height and weight. BMI was calculated by dividing weight in kilogrammes by square of height in metres. Body composition, including body fat mass (BFM), fat free mass (FFM), skeletal muscle mass (SMM), visceral fat area (VFA), and skeletal muscle index (SMI), was measured by the direct segmental multi‐frequency bioelectrical impedance analysis (BIA) method (Inbody 770, Biospace Co., BR‐Chinese‐C7‐B‐140218), which is a relatively simple and harmless yet reliable and popular tool for assessing body composition currently. VSR is the ratio of VFA to SMM. The participants were required to stand upright in bare feet with their arms straight and hold the handles of the analyser, so that their palms, thumbs, heels, and soles were all in full contact with an 8‐pole tactile electrode. Then, the measurement began and the data were automatically saved in the computer once the measurement was completed.

### 2.3 Clinical and biochemical measurement

Past medical histories and demographic characteristics, including age and gender, were recorded in detail for all of the participants. After a 10‐min rest, blood pressure was measured for three times with an automatic electronic blood pressure monitor (AC‐05C, Ling Qian) and the mean value was taken. Uniformly trained ultrasound physicians with ultrasound diagnosis systems (Philips, Phoenix and Neusoft Medical Systems Co., Ltd.) performed abdominal ultrasound examination to provide the imaging basis for the diagnosis of MAFLD. All blood samples were collected in the morning after an overnight fast, centrifuged as soon as possible, and stored at −80°C for subsequent detection assays. Serum uric acid (SUA), fasting plasma glucose (FPG), total cholesterol (TC), triglycerides (TG), low‐density lipoprotein cholesterol (LDL‐C), and high‐density lipoprotein cholesterol (HDL‐C) levels were determined using an automatic biochemical analyser (TBA120FR, Toshiba).

### 2.4 Definitions of cardiometabolic diseases

Hepatic steatosis was established when two or more of the following requirements given in parentheses were met according to the abdominal ultrasonography (diffuse enhanced echo of liver with liver echogenicity greater than kidney or spleen, deep attenuation of ultrasound signal, and vascular blurring). MAFLD was diagnosed as hepatic steatosis with overweight/obesity, T2DM or evidence of metabolic dysregulation.^8^ Hyperglycemia was defined as FPG ≥7.0 mmol/L or previously diagnosed as diabetes. Hypertension was defined as systolic blood pressure (SBP) ≥140 mmHg and/or diastolic blood pressure (DBP) ≥90 mmHg or previously diagnosed as hypertension. Dyslipidemia was defined as TC ≥6.2 mmol/L or LDL‐C ≥4.1 mmol/L or HDL‐C <1.0 mmol/L or TG ≥2.3 mmol/L based on the Chinese guidelines for the management of dyslipidemia in adults (2016)^9^ or previously diagnosed as dyslipidemia. Hyperuricemia was defined as SUA ≥420 μmol/L according to the guideline for the diagnosis and treatment of hyperuricemia and gout in China (2019)^10^ or previously diagnosed as hyperuricemia or gout.

### 2.5 Calculation of the prevalence of cardiometabolic diseases

The prevalence of MAFLD, hyperglycemia, hypertension, dyslipidemia, and hyperuricemia was calculated by the number of patients with MAFLD, hyperglycemia, hypertension, dyslipidemia, and hyperuricemia in the study population divided by the total number of people in the study population.

### 2.6 Statistical analysis

For continuous variables, Kolmogorov‐Smimov test was first performed to evaluate whether the data were normally distributed or not. Continuous variables of normal and non‐normal distribution were expressed as mean ± standard deviation and median (interquartile range), respectively. Categorical variables were presented as percentage or frequency. Continuous data were analysed using the independent two‐sample *t* test or the Mann–Whitney *U* test. Homogeneity of variances was tested by Levene's Test. Chi‐squared test was performed for analysis of categorical variables. One‐way ANOVA was performed to confirm there were statistical differences of VSR among different age groups, and then Bonferroni post‐hoc test was used to compare the specific differences among groups. Binary logistic regression analysis was adopted to estimate the odds ratio (OR) and 95% confidence interval (CI) between VSR and MAFLD, hyperglycemia, hypertension, dyslipidemia, and hyperuricemia after it was confirmed that the regression model could fit the data correctly by Hosmer–Lemeshow test. One‐way ANOVA was used to verify whether there were non‐linear associations between VSR and cardiometabolic diseases and then restricted cubic splines were used to evaluate the non‐linear relationships between VSR and the risk of cardiometabolic diseases. Knots were placed at 5th, 27.5th, 50th, 72.5th, and 95th percentiles. All statistical analyses were performed using SAS version 9.4 for Windows (SAS Institute). *p* value < 0.05 was considered to be statistically significant.

## 3 RESULTS

### 3.1 Clinical characteristics of the study population

A total of 5158 participants were recruited in this study, consisting of 2154 men (41.8%) and 3004 women (58.2%). The clinical characteristics of the study population are presented in Table 1. The average age of all the participants was 44.54 ± 14.71 years old, indicating that this was mainly a young and middle‐aged population. The overall prevalence of MAFLD, dyslipidemia, hypertension, hyperuricemia, and hyperglycemia was 30.9%, 27%, 25.4%, 12.6%, and 6.0%, respectively. BMI and some parameters of body composition, including BFM, FFM, SMM, and SMI were significantly higher in male than in female (*p* < 0.001). The percentages of FFM and SMM were significantly higher in male than in female, and the percentage of BFM was significantly higher in female than in male (*p* < 0.001). VFA was obviously higher in female than in male (*p* = 0.002), displaying an absolutely opposite trend to that of BMI. Women had relatively high VFA and low SMM, so VSR was significantly higher in women than in men (*p* < 0.001). The prevalence of MALFD, hyperglycemia, hypertension, dyslipidemia, and hyperuricemia was higher in male than in female (*p* < 0.001).

**TABLE 1:** Characteristics of the study population according to sex

Characteristics	Total (*N* = 5158)	Males (*N* = 2154)	Females (*N* = 3004)	*p*
Age (years)	44.54 ± 14.71	45.48 ± 15.0	43.86 ± 14.47	<0.001**
Height (cm)	165.46 ± 8.31	172.36 ± 6.31	160.51 ± 5.6	<0.001**
Weight (kg)	66.78 ± 13.17	76.71 ± 11.58	59.65 ± 8.95	<0.001**
BMI (kg/m^2^)	24.26 ± 3.63	25.78 ± 3.36	23.17 ± 3.41	<0.001**
SBP (mmHg)	122.66 ± 17.88	127.91 ± 16.45	118.89 ± 17.91	<0.001**
DBP (mmHg)	77.46 ± 10.56	80.66 ± 10.53	75.16 ± 9.97	<0.001**
FPG (mmol/L)	5.14 (4.83–5.56)	5.25 (4.92–5.76)	5.06 (4.77–5.43)	<0.001**
TC (mmol/L)	5.08 ± 0.97	5.02 ± 0.91	5.12 ± 1.0	<0.001**
TG (mmol/L)	1.13 (0.81–1.63)	1.35 (0.99–1.91)	1.0 (0.74–1.42)	<0.001**
HDL‐C (mmol/L)	1.55 ± 0.38	1.39 ± 0.3	1.67 ± 0.38	<0.001**
LDL‐C (mmol/L)	2.71 ± 0.58	2.69 ± 0.55	2.73 ± 0.61	0.004*
SUA (umol/L)	311.02 ± 82.31	368.2 ± 74.72	270.03 ± 60.06	<0.001**
BFM (kg)	20.41 ± 6.46	20.99 ± 6.8	19.99 ± 6.18	<0.001**
FFM (kg)	46.35 ± 9.66	55.68 ± 6.95	39.66 ± 4.29	<0.001**
SMM (kg)	25.45 ± 5.87	31.15 ± 4.19	21.35 ± 2.56	<0.001**
VFA (cm^2^)	95.07 ± 36.01	93.23 ± 34.35	96.38 ± 37.1	0.002*
VSR (cm^2^/kg)	3.88 ± 1.62	3.00 ± 1.06	4.51 ± 1.66	<0.001**
SMI (kg/m^2^)	6.97 ± 1.08	7.98 ± 0.7	6.25 ± 0.61	<0.001**
MAFLD (%)	1594 (30.9)	948 (44.0)	646 (21.5)	<0.001**
Hyperglycemia (%)	312 (6.0)	185 (8.6)	127 (4.2)	<0.001**
Hypertension (%)	1309 (25.4)	746 (34.6)	563 (18.7)	<0.001**
Dyslipidemia (%)	1394 (27.0)	669 (31.1)	725 (24.1)	<0.001**
Hyperuricemia (%)	648 (12.6)	506 (23.5)	142 (4.7)	<0.001**

*Note*: *p* values marked with ‘*’ indicate significant differences (*p* < 0.05) between males and females. *p* values marked with ‘**’ indicate significant differences (*p* < 0.001) between males and females.

Abbreviations: BFM, body fat mass; BMI, body mass index; DBP, diastolic blood pressure; FFM, fat free mass; FPG, fasting plasma glucose; HDL‐C, high‐density lipoprotein cholesterol; LDL‐C, low‐density lipoprotein cholesterol; MAFLD, metabolic associated fatty liver diseases; SBP, systolic blood pressure; SMI, skeletal muscle index; SMM, skeletal muscle mass; SUA, serum uric acid; TC, total cholesterol; TG, triglycerides; VFA, visceral fat area; VSR, visceral fat area to skeletal muscle mass ratio.

### 3.2 Prevalence of cardiometabolic diseases according to VSR quartiles in male and female

**FIGURE 2:**
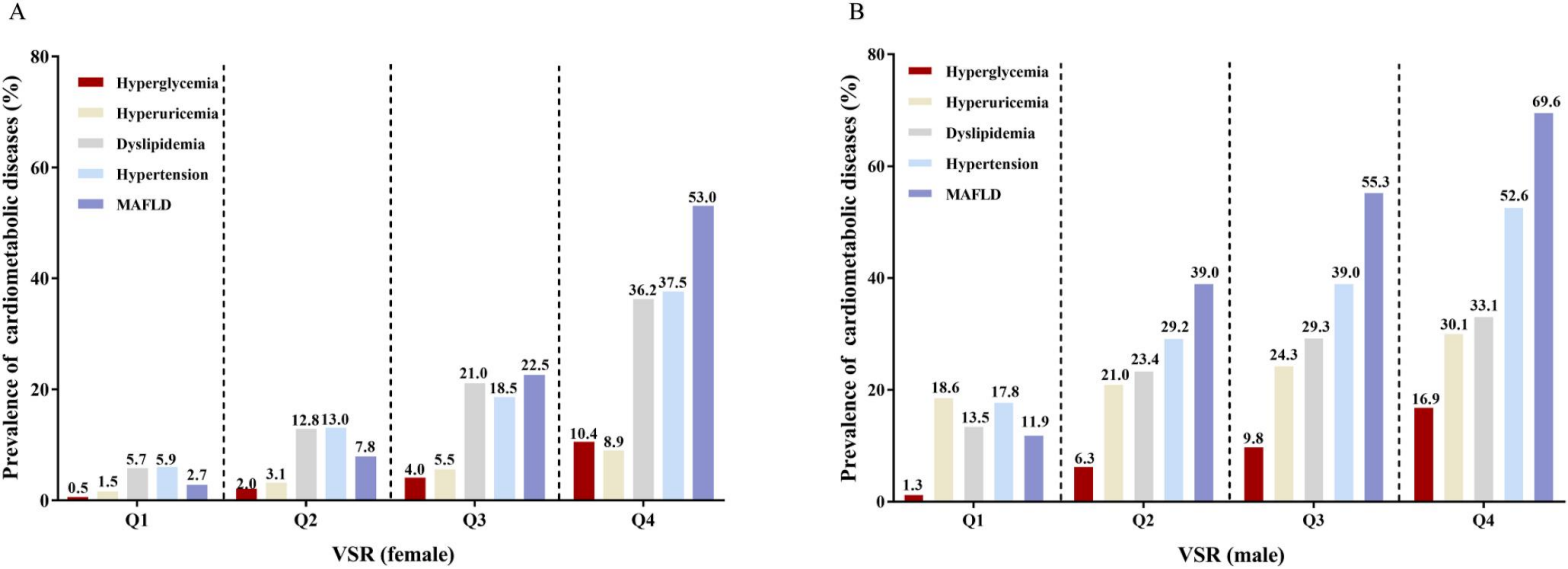
Prevalence of cardiometabolic diseases grouped by VSR quartiles. (A) Prevalence of cardiometabolic diseases grouped by VSR quartiles in female. (B) Prevalence of cardiometabolic diseases grouped by VSR quartiles in male. Quartiles of VSR in male, Q1: 0.35–2.28, Q2: 2.29–2.86, Q3: 2.87–3.56, Q4: 3.57–8.84; quartiles of VSR in female, Q1: 1.24–3.16, Q2: 3.17–4.27, Q3: 4.28–5.59, Q4: 5.60–11.32. MAFLD, metabolic associated fatty liver disease; VSR, visceral fat area to skeletal muscle mass ratio.

According to the VSR quartiles, men and women were divided into four subgroups, respectively. The prevalence of MAFLD, hyperglycemia, hypertension, dyslipidemia and hyperuricemia was calculated in the four subgroups of both genders separately. Results showed that with the increase of VSR, the prevalence of the five cardiometabolic conditions rose up both in male and female. Compared with participants with VSR in the other three quartiles, participants with VSR in the fourth quartile had the highest prevalence of the above‐mentioned cardiometabolic diseases (*p* < 0.001), regardless of gender. Moreover, in each of the same quartile, the prevalence of cardiometabolic diseases in male was higher than in female (*p* < 0.001). The prevalence of MAFLD was the highest among the five cardiometabolic diseases (*p* < 0.001). The results are exhibited in Figure 2.

### 3.3 Associations between VSR and age

**FIGURE 3:**
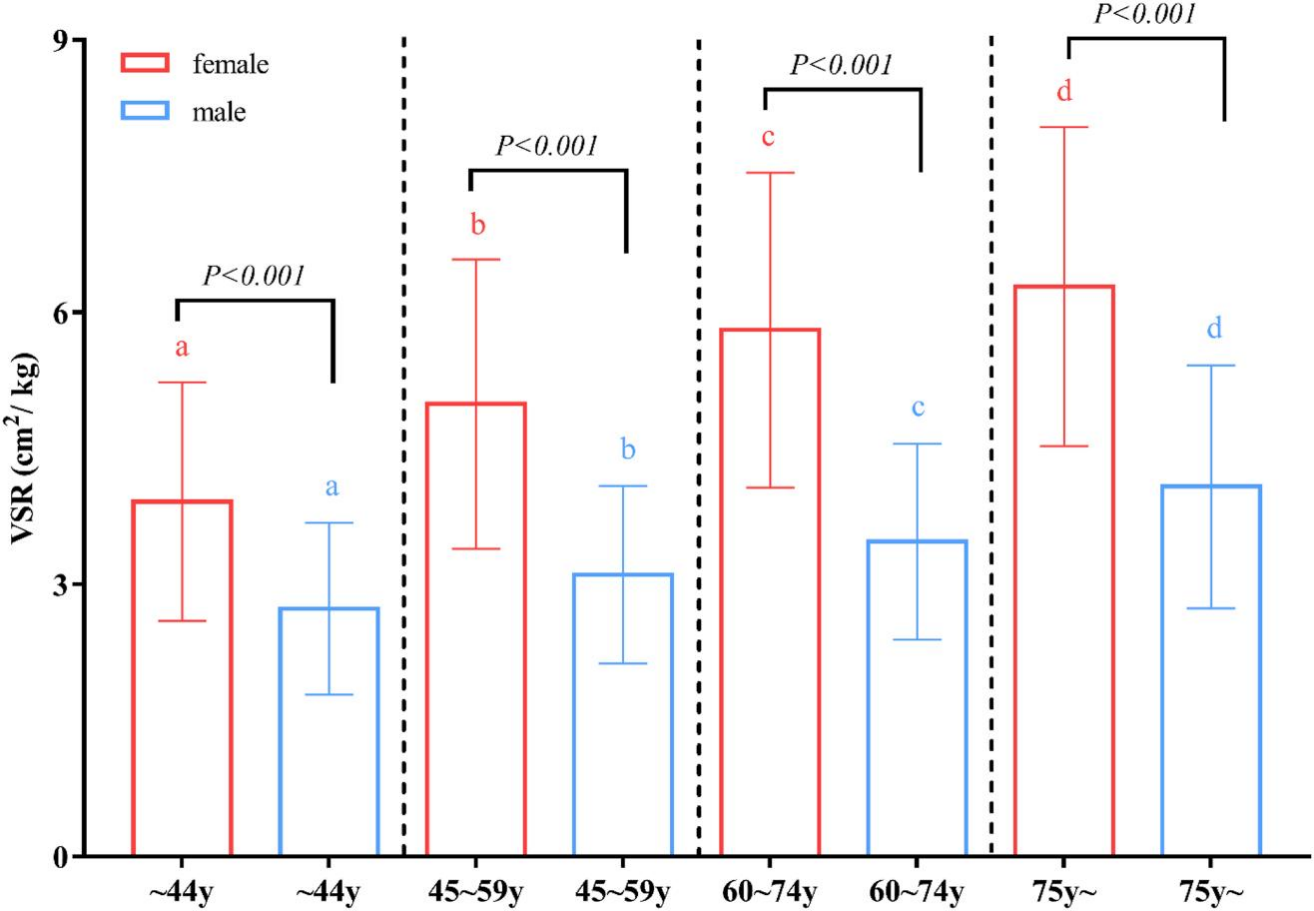
VSR in different age groups of male and female. The four age groups were ≤44, 45–59, 60–74 and ≥75 years old. a, b, c, and d indicated there were significant differences among the four age groups (*p* < 0.05). VSR, visceral fat area to skeletal muscle mass ratio.

In order to evaluate the associations between VSR and age, men and women were divided into four age groups (≤44, 45–59, 60–74 and ≥75 years old). VSR in different age groups of male and female were investigated and the results are demonstrated in Figure 3. There were significant differences of VSR among the four age groups both in male and female and VSR increased significantly with age (*p* < 0.05). In the same age group, VSR in female was significantly higher than in male (*p* < 0.001).

### 3.4 Associations between VSR and cardiometabolic diseases

The associations of VSR with cardiometabolic diseases were explored by binary logistic regression analysis and the results are shown in Table 2. We could see that in both genders, VSR was positively correlated with the five cardiometabolic diseases; with the increase of VSR by one quartile, the ORs increased significantly for all the five cardiometabolic diseases (*p*_trend_ < 0.001). With regard to the highest versus the lowest quartile, the ORs for MAFLD, hyperglycemia, hypertension, dyslipidemia, and hyperuricemia were 17.23 (95% CI, 12.52–23.71), 15.47 (95% CI, 7.1–33.72), 5.12 (95% CI, 3.88–6.76), 3.16 (95% CI, 2.33–4.28), and 1.89 (95% CI, 1.42–2.51) in male, respectively. And in female, the corresponding ORs were as follows, 41.15 (95% CI, 25.80–65.63), 21.62 (95% CI, 7.87–59.36), 9.64 (95% CI, 6.88–13.53), 9.34 (95% CI, 6.63–13.14), and 6.58 (95% CI, 3.45–12.56). The non‐linear relationships of VSR with cardiometabolic diseases in both genders were further explored by restricted cubic splines. The results showed there were significant positive non‐linear relationships between VSR and the risk of MAFLD, dyslipidemia, hyperglycemia, and hypertension in both genders (*p* for non‐linearity <0.05). The risk was relatively flat until when VSR reached 3.078 cm^2^/kg in men and 4.750 cm^2^/kg in women and then started to increase rapidly afterwards. In men, however, the risk slowed down after VSR value got to around 4 cm^2^/kg and the curve became relatively flat and even tended to decline. The non‐linear correlations of VSR with hyperuricemia were not statistically significant in both genders, but the previous binary logistic regression analysis had shown significant positive associations between VSR and hyperuricemia (*p*_trend_ < 0.001). The results are shown in Figure 4.

**TABLE 2:** OR with 95% CI for associations between VSR and cardiometabolic diseases according to sex

	VSR (cm^2^/kg)	*N*	MAFLD	Hyperglycemia	Hypertension	Dyslipidemia	Hyperuricemia
Males	Q1*	539	1 (ref.)	1 (ref.)	1 (ref.)	1 (ref.)	1 (ref.)
	Q2	538	4.75 (3.47, 6.50)	5.13 (2.25, 11.67)	1.90 (1.42, 2.54)	1.95 (1.42, 2.68)	1.17 (0.86, 1.58)
	Q3	539	9.18 (6.72, 12.53)	8.29 (3.73, 18.4)	2.95 (2.23, 3.90)	2.65 (1.94, 3.60)	1.41 (1.05, 1.89)
	Q4	538	17.23 (12.52, 23.71)	15.47 (7.1, 33.72)	5.12 (3.88, 6.76)	3.16 (2.33, 4.28)	1.89 (1.42, 2.51)
	*p*_trend_		<0.001**	<0.001**	<0.001**	<0.001**	<0.001**
Females	Q1	751	1 (ref.)	1 (ref.)	1 (ref.)	1 (ref.)	1 (ref.)
	Q2	751	3.10 (1.85, 5.21)	3.80 (1.25, 11.49)	2.40 (1.66, 3.49)	2.41 (1.65, 3.50)	2.12 (1.03, 4.38)
	Q3	751	10.60 (6.58, 17.06)	7.76 (2.72, 22.14)	3.64 (2.55, 5.20)	4.38 (3.07, 6.24)	3.88 (1.98, 7.61)
	Q4	751	41.15 (25.80, 65.63)	21.62 (7.87, 59.36)	9.64 (6.88, 13.53)	9.34 (6.63, 13.14)	6.58 (3.45, 12.56)
	*p*_trend_		<0.001**	<0.001**	<0.001**	<0.001**	<0.001**

*Note*: *p*_trend_ values marked with ‘**’ indicate significant differences (*p*_trend_ < 0.001) between VSR and cardiometabolic diseases. Quartiles of VSR in males, Q1: 0.35–2.28, Q2: 2.29–2.86, Q3: 2.87–3.56, Q4: 3.57–8.84; quartiles of VSR in females, Q1: 1.24–3.16, Q2: 3.17–4.27, Q3: 4.28–5.59, Q4: 5.60–11.32.

Abbreviations: CI, confidence interval; MAFLD, metabolic associated fatty liver disease; OR, odds ratio; VSR, visceral fat area to skeletal muscle mass ratio.

FIGURE 4Non‐linear relationships between VSR and the risk of cardiometabolic diseases. (A) Non‐linear relationships between VSR and the risk of MAFLD in male. (B) Non‐linear relationships between VSR and the risk of dyslipidemia in male. (C) Non‐linear relationships between VSR and the risk of hyperglycemia in male. (D) Non‐linear relationships between VSR and the risk of hypertension in male. (E) Non‐linear relationships between VSR and the risk of hyperuricemia in male. (F) Non‐linear relationships between VSR and the risk of MAFLD in female. (G) Non‐linear relationships between VSR and the risk of dyslipidemia in female. (H) Non‐linear relationships between VSR and the risk of hyperglycemia in female. (I) Non‐linear relationships between VSR and the risk of hypertension in female. (J) Non‐linear relationships between VSR and the risk of hyperuricemia in female. Data were OR (solid line) and 95% CI (shadow area) from logistic regression analysis with restricted cubic splines. CI, confidence intervals; MAFLD, metabolic associated fatty liver disease; OR, odds ratio; VSR, visceral fat area to skeletal muscle mass ratio.

**Figure:**
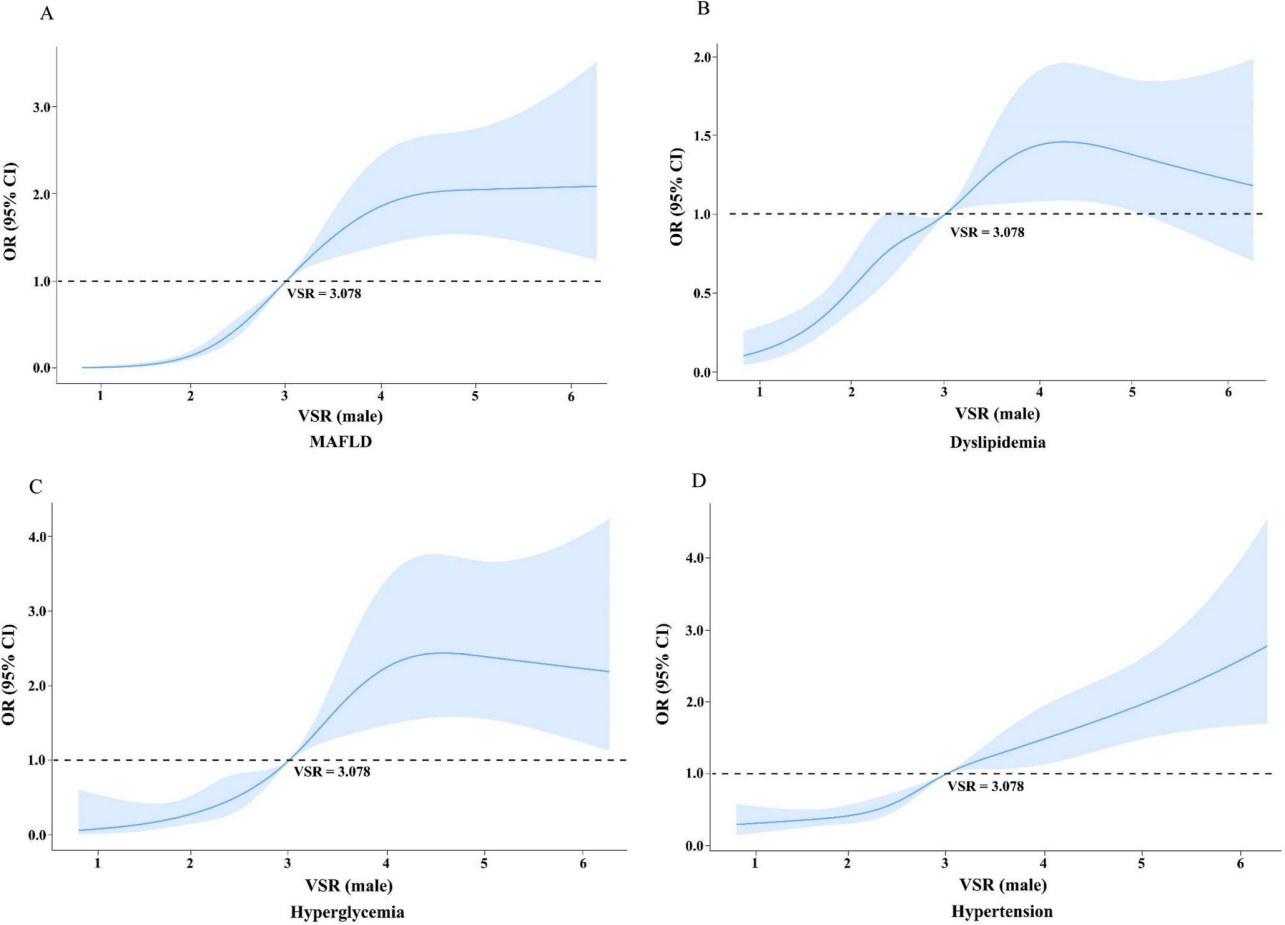

**Figure:**
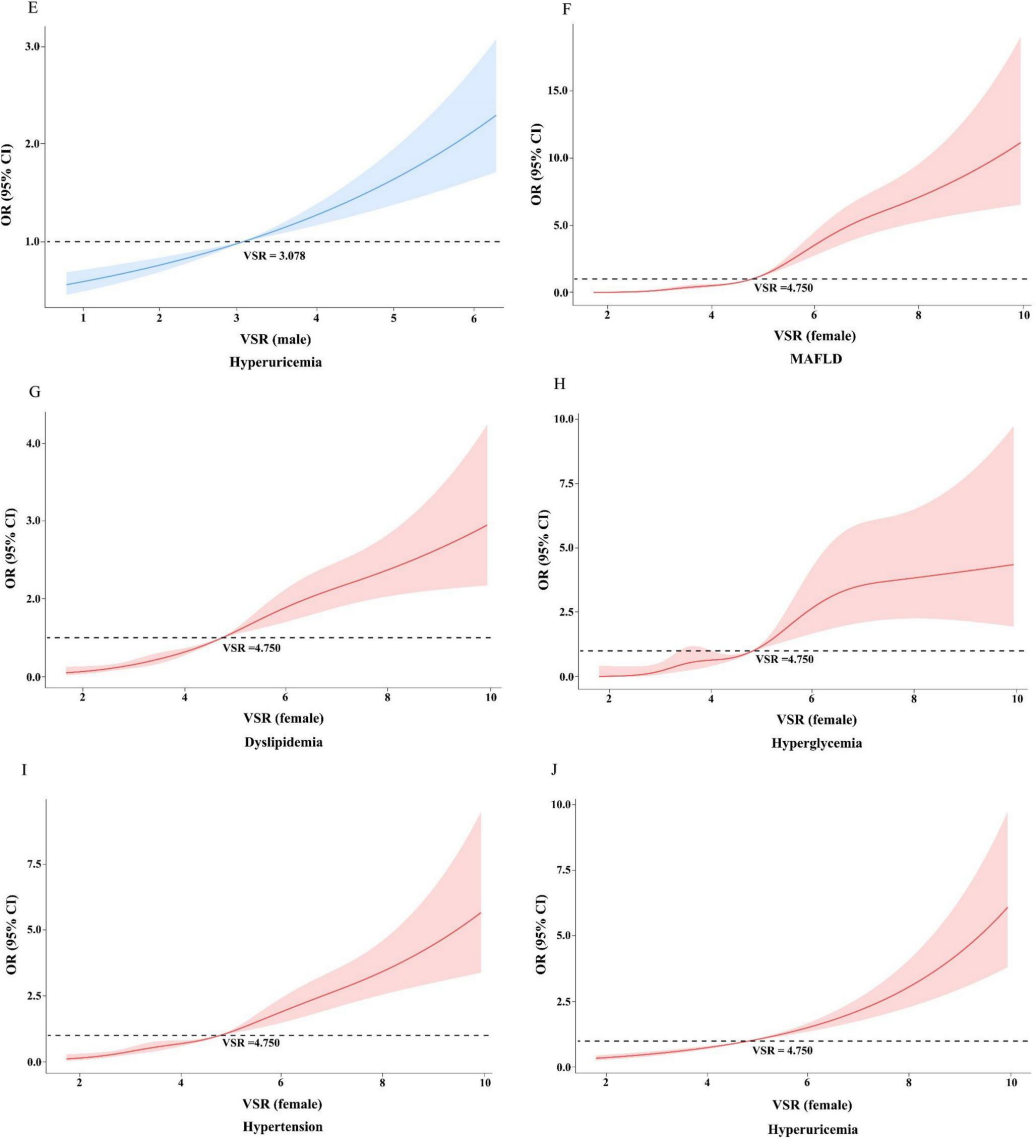

## 4 DISCUSSION

In the current study, we investigated the associations between increased VSR and the risk of a constellation of common cardiometabolic diseases. The results showed that increased VSR was positively associated with cardiometabolic diseases regardless of gender. In addition, as VSR increased, the risk of cardiometabolic diseases was significantly higher in women than in men.

Numerous previous studies have shown that obesity, especially visceral obesity, is a highly common feature of insulin resistance and its associated cardiometabolic diseases.^11^, ^12^ The mechanisms by which visceral obesity leads to cardiometabolic diseases are not yet fully understood. Recent studies have found that visceral obesity may contribute to cardiometabolic diseases by a series of biological pathways: (1) increased adipose tissue deposited in the viscera can secrete a large number of inflammatory cytokines such as interleukin (IL)‐6, tumour necrosis factor (TNF)‐α, and IL‐1β, which result in a low‐grade inflammatory state, insulin resistance and eventually cardiometabolic diseases^13^; (2) inflamed adipose tissue significantly reduces the production and secretion of adiponectin which has anti‐lipotoxic and insulin sensitising effects on pancreatic β‐cells and hepatocytes^14^; (3) the hyperlipolytic activity of adipose tissue causes the release of nonesterified fatty acid (NEFA) into the circulation and delivery to liver to increase, which promotes hepatic β‐oxidation and insulin resistance^15^; (4) exosomes derived from obese adipose tissue macrophages can cause insulin resistance.^16^

Skeletal muscle loss also plays a vital role in insulin resistance and cardiometabolic diseases. Data from The Third National Health and Nutrition Examination Survey in America found that higher muscle mass was inversely associated with insulin resistance and the risk of prediabetes.^17^ Jennifer et al. investigated the associations of low muscle volume with metabolic comorbidities in patients with non‐alcoholic fatty liver disease in the UK Biobank study, and the results showed that low muscle volume was linked to a high prevalence of T2DM and coronary heart disease.^18^ The protective effect of skeletal muscle against cardiometabolic diseases is due to the fact that it is the primary tissue contributing to insulin‐mediated postprandial glucose uptake.^19^ In addition, skeletal muscle is an endocrine organ that can produce and secrete myokines such as irisin and myonectin.^20^ Irisin and myonectin are important myokines that have been proven to facilitate adipose tissue browning, promote thermogenesis, and increase the uptake and oxidation of NEFA in the liver and adipose tissue.^21^, ^22^ Therefore, impaired myokines secretion resulting from skeletal muscle loss may play a role in the development of insulin resistance and cardiometabolic diseases.^23^

Given the detrimental roles of increased visceral fat and decreased skeletal muscle in the pathogenesis of cardiometabolic diseases, thus VSR, which was calculated as the ratio of VFA to SMM, was positively associated with an increased risk of cardiometabolic diseases.

As for the gender differences in the association of VSR with cardiometabolic diseases, we found that the risk of cardiometabolic diseases was higher in women than in men as VSR increased. Similar results were found in previous studies, which had shown that compared with men, women had a greater risk of cardiometabolic diseases, such as MAFLD, diabetes, cardiovascular disease, and hypertriglyceridemia.^24^, ^25^ The potential mechanisms for the gender‐specific differences may be multifactorial, including genetic, biological, and environmental factors.^24^ The biological mechanisms have mainly centred on the differences in fat distribution and mass of skeletal muscle caused by different sex hormone levels between men and women.^26^ Unlike men being prone to have abdominal obesity, fat tends to accumulate in the lower body in women. Therefore, when visceral fat increases, women are at higher risk of cardiometabolic diseases compared with men. Women may be protected from atherosclerotic cardiovascular disease, MAFLD, and other cardiometabolic diseases due to estrogen before menopause.^27^, ^28^, ^29^ When women are in the postmenopausal stage, the risk of cardiometabolic diseases is further elevated for the protective effect of estrogen has been lost during this stage.^30^ On the other hand, some studies have found that higher levels of circulating testosterone in men are associated with a decreased risk of cardiovascular diseases due to the beneficial effects of testosterone on promoting muscle mass and strength, increasing cardiac output and inhibiting myofibroblast proliferation.^31^, ^32^ Women have lower testosterone levels, muscle mass and strength than men, which may be one of the reasons why women have greater risk of cardiometabolic diseases compared with men.

This study was conducted in a natural population undergoing regular health check‐ups. The mean age of all the participants was around 44 years old, indicating that this study population was mainly composed of middle‐aged individuals and was the backbone of social production and development. However, the prevalence of the five cardiometabolic diseases in this population was alarming. The prevalence rates of MAFLD, dyslipidemia, hypertension, and hyperuricemia were similar to those found in previous epidemiological studies in people of Chinese ethnicity, except for hyperglycemia.^33^, ^34^, ^35^ According to the latest epidemiological survey, the prevalence of diabetes in China is around 12.8%,^36^ which is much higher than the result of our study. This discrepancy may be attributed to the fact that some patients with T2DM may have elevated postprandial blood glucose at the early stage of the disease, but their FPG can still remain in the normal range.^37^ The data in this study came from a population receiving their annual health check‐ups, with only one fasting blood sample taken and FPG measured, which might miss some patients with diabetes. In addition, participants recruited in this study were mainly young and middle‐aged people, which might also lead to the relatively low prevalence of hyperglycemia.

There are several strengths in our study. First of all, this is the first analysis to examine the associations of the joint effect of visceral fat and skeletal muscle mass on cardiometabolic disorders in a large natural population of Chinese ethnicity. Secondly, this study has clarified gender differences in these associations based on the significant differences in body composition in men and women. Thirdly, we have used restricted cubic spline curves to clearly visualise the non‐linear relationships between VSR and cardiometabolic diseases when they are not linearly related. The study also has several limitations. Firstly, this is a cross‐sectional study, which cannot prove the causality between VSR and cardiometabolic diseases. Secondly, information on physical activity levels and medication that might have influenced the results was not collected. Thirdly, the participants enroled in this study were those who received their annual health check‐up, and only their fasting blood samples could be obtained, which might lead to the missed diagnosis of some patients with hyperglycemia.

In conclusion, our results suggested that VSR was positively associated with cardiometabolic diseases regardless of gender. As VSR increased, the risk of cardiometabolic diseases in women was significantly higher than in men. These results indicated that women should be more alert to the risk of cardiometabolic diseases caused by the increase of VSR than men. Well‐designed, prospective cohort studies, including larger sample sizes, and other age and ethnic groups, are needed to further confirm and replicate our conclusions.

## AUTHOR CONTRIBUTIONS

Shi Zhang analysed and interpreted the patient data and was the major contributor in writing the manuscript; Yaping Huang analysed and interpreted the data; Jing Li, Xincheng Wang, Minying Zhang, Yanju Zhang, and Meiyang Du assisted with data collection; Xiaohe Wang assisted with data analyses; Jingna Lin and Chunjun Li contributed to the design of the study. All authors have read and approved the final manuscript.

## CONFLICT OF INTEREST

The authors declare they have no conflict of interest.

### PEER REVIEW

The peer review history for this article is available at https://publons.com/publon/10.1002/dmrr.3597.

## ETHICS STATEMENT

The study was approved by the Medical Ethics Committee of Tianjin Union Medical Center (No. 2021C06) and all participants provided written informed consents.

## Data Availability

The data analysed during the current study is available from the corresponding author upon reasonable request.
